# Supplementary material for: The Canadian collaborative project on genetic susceptibility to multiple sclerosis cohort population structure and disease etiology
Source: Front Neurol. 2025 Mar 5;16:1509371. doi: 10.3389/fneur.2025.1509371 (PMC11919664; doi:10.3389/fneur.2025.1509371)
Supplement: Supplementary file 2 [file Data_Sheet_1.pdf]

Supplementary Material

1 Supplementary Figures

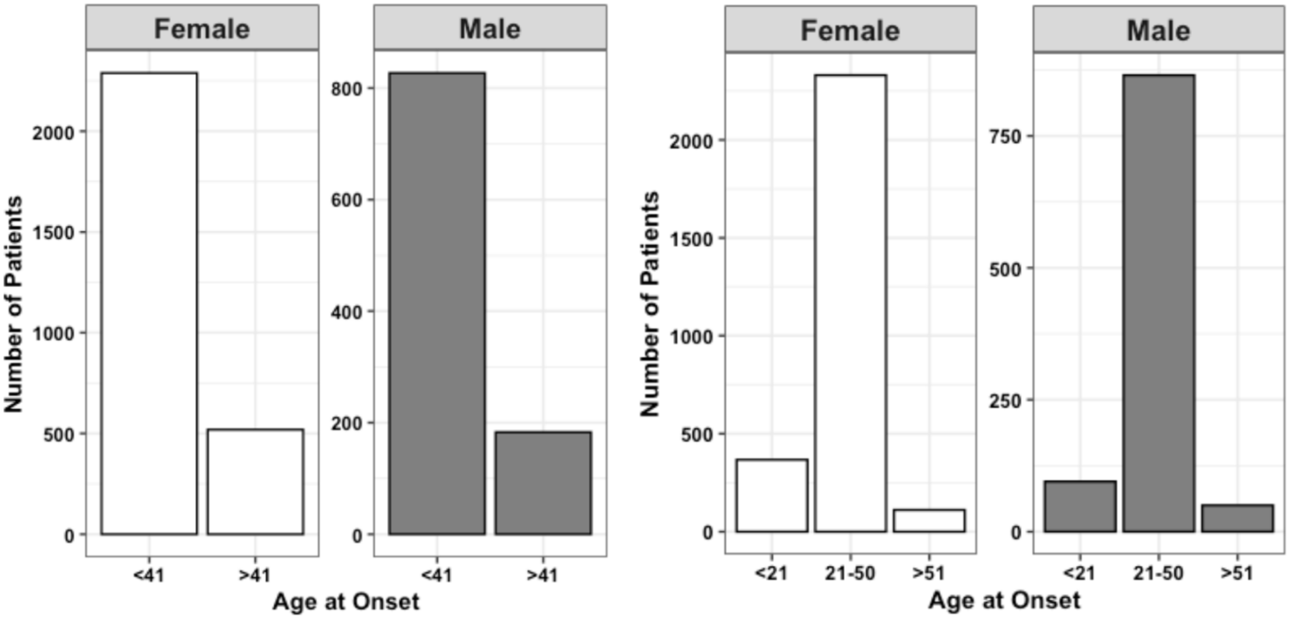

Supplementary Figure 1. Age of MS onset distribution by sex (n=3,787). Data represent all patients for whom age at initial diagnosis was provided.

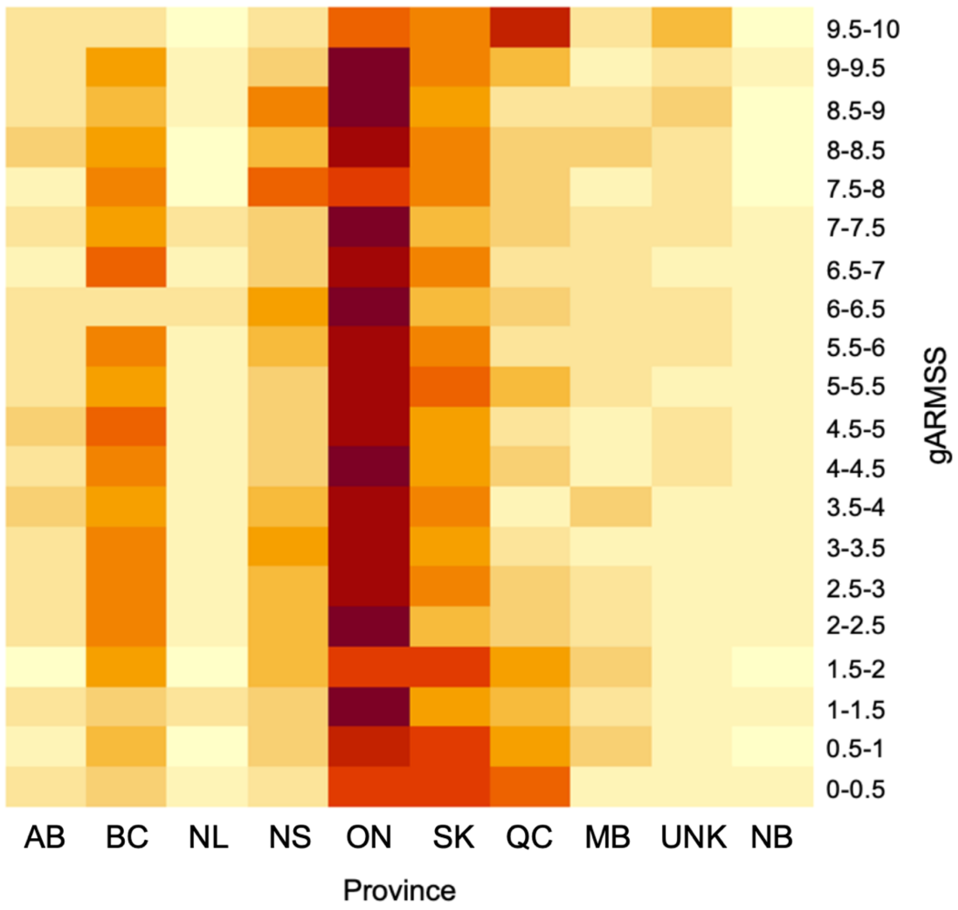

Supplementary Figure 2. Heatmap distribution of ARMSS scores for proband patients by province (n=2,473). Darker shading indicates higher incidence of a particular gARMSS score stratum in a particular province. AB, Alberta; BC, British Columbia; NL, Newfoundland; NS, Nova Scotia; ON, Ontario; SK, Saskatchewan; QC, Quebec; MB, Manitoba; UNK, Unknown; NB, New Brunswick. gARMSS, global age-related MS severity
